# Hepatic Abscess With Clostridium perfringens Bacteremia Leading to Hemolysis: A Case Report

**DOI:** 10.7759/cureus.84242

**Published:** 2025-05-16

**Authors:** Joel Collins II, Katelyn Courtney, James Espinosa, Henry Schuitema, Alan Lucerna

**Affiliations:** 1 Emergency Medicine, Jefferson Health NJ, Stratford, USA

**Keywords:** acalculous cholecystitis, clostridium perfringens, clostridium perfringens bacteremia, clostridium perfringens infection and hemodialysis, hepatic abscess

## Abstract

We describe the case of a 64-year-old male who presented with acute acalculous cholecystitis complicated by the formation of a hepatic abscess. Blood cultures subsequently grew *Clostridium perfringens*, and culture of the aspirated hepatic abscess fluid confirmed its presence.* *The clinical course was further complicated by severe intravascular hemolysis, leading to acute renal failure that required temporary hemodialysis.

Management included the prompt initiation of broad-spectrum intravenous antibiotic therapy in conjunction with image-guided percutaneous drainage of the hepatic abscess. *C. perfringens* sepsis is associated with high morbidity and mortality, primarily due to its rapid progression and toxin-mediated hemolysis. This case underscores the importance of early recognition, aggressive antimicrobial therapy, and timely source control to optimize clinical outcomes.

## Introduction

*Clostridium perfringens* is the pathogen responsible for a multitude of infections, including necrotizing soft tissue infections, bacteremia, and life-threatening hemolysis. As a general principle, management should include removal or control of the source through cholecystectomy or percutaneous drains as well as treatment with broad-spectrum antibiotics [1]. 

Sepsis caused by *C. perfringens* is associated with a high mortality rate [1,2]. *C. perfringens* is classified into types A, B, C, D, and E based on the toxins it produces [2,3]. Type A, which produces alpha toxin, is the most clinically significant. It is responsible for conditions such as gas gangrene, gastrointestinal infections, and severe intravascular hemolysis [2]. In approximately 7-15% of *C. perfringens* bacteremia cases, Type A can cause massive, often fatal intravascular hemolysis [1,4]. The alpha toxin exhibits strong phospholipase C activity, leading to erythrocyte lysis [5]. The rapid growth rate of *C. perfringens*, with an approximate doubling time of seven minutes, contributes significantly to the rapid clinical deterioration seen in affected patients, underscoring the importance of early recognition and diagnosis [6]. Immunocompromised individuals are particularly susceptible to *C. perfringens* infections [7].

This case report was presented in poster form at the Rowan University Research Day, Stratford NJ, on May 2, 2024.

## Case presentation

We present the case of a 64-year-old man who arrived at the emergency department (ED) with right upper quadrant abdominal pain. He also reported right-sided chest pain, cough, and a fever of 102.0°F (38.89°C) at home. Notably, he had experienced a similar episode of acute right upper quadrant pain approximately one month prior. At that time, a diagnostic evaluation, including a computed tomography angiography (CTA) of the chest and a CT scan of the abdomen and pelvis, was unremarkable. Approximately nine days before the current presentation, the patient had undergone a total right knee arthroplasty. The current abdominal pain was described as sharp, rated 10 out of 10 in intensity, and was accompanied by a single episode of non-bilious, non-bloody diarrhea.

His past medical history included deep vein thrombosis with Factor V Leiden, hypertension, hyperlipidemia, and gastroesophageal reflux disease.

On presentation, vital signs were as follows: heart rate 99 beats per minute, respiratory rate 18 breaths per minute, blood pressure 143/76 mmHg, and temperature 102.0°F (38.89°C). His pain score remained 10 out of 10. Physical examination revealed right upper quadrant tenderness without rigidity, guarding, or rebound; however, Murphy’s sign was positive. The remainder of the examination was unremarkable.

Blood cultures and serum lactate were obtained. The patient received a 30 cc/kg fluid bolus of normal saline based on ideal body weight. Empiric intravenous antibiotics were initiated, including metronidazole, piperacillin/tazobactam, and vancomycin. Intravenous morphine sulfate was administered for pain control.

Laboratory evaluation revealed a markedly elevated white blood cell count of 49,000 cells/μL, hemoglobin of 7.3 g/dL, and a creatinine level of 1.7 mg/dL, slightly above baseline. Total bilirubin was elevated at 19.4 mg/dL (reference range 0.0-1.0 mg/dL), with direct bilirubin at 13.9 mg/dL (reference range 0.0-0.3 mg/dL). Liver enzymes were significantly elevated: AST at 3,904 IU/L (reference range 0-37 IU/L) and ALT at 1,933 IU/L (reference range 0-40 IU/L). Alkaline phosphatase was also elevated at 183 IU/L (reference range 39-117 IU/L). No schistocytes were seen on the peripheral smear (Table 1).

**Table 1:** Laboratory values in the emergency department

Laboratory results	Result	Normal range	Units
White blood cell count	49,000	4,000 to 11,000	cells/uL
Hemoglobin	7.3	10.6-15.6	g/dL
Platelet count	180	150-400	K/uL
Sodium	137	135-154	mEq/L
Potassium	3.6	3.5-5	mEq/L
BUN	18.0	5 to 20	mg/dL
Creatinine	1.7	0.6-1.2	mg/dL
Glucose	95.0	70-100	mg/dL
Calcium	8.7	8.5-10.5	mg/dL
Chloride	101.0	95-105	mEq/L
Bicarbonate	24.0	23-29	mEq/L
Magnesium	1.8	1.7-2.2	mg/dL
lactate	4.1	0.5-2.2	mmol/L
PT	11.0	11-13.5	sec
PTT	33.0	25-35	sec
INR	1.0	0.8-1.1	INR ratio
Total bilirubin	19.4	0.1 to 1.2	mg/dL
Direct bilirubin	13.9	0 to 0.3	mg/dL
AST	3,904	8 to 33	IU/L
ALT	1,933	7 to 56	IU/L
Alkaline phosphatase	183	39 to 117	IU/L
Blood culture	Clostridium perfringens	Negative	NA
Urine color	Clear	Yellow	NA
Urine clarity	Clear	Clear	NA
Urine specific gravity	1.0	1.005-1.030	NA
Urine pH	7.0	5 to 7.5	NA
Urine glucose	Negative	Negative	NA
Urine protein	Negative	Negative	NA
Urine bilirubin	Positive	Negative	NA
Urine urobilinogen	Positive	Negative	NA
Urine ketones	Negative	Negative	NA
Urine blood	Negative	Negative	NA
Urine white cells	Negative	0-5/HPF	cells/HPF
Urine red cells	Negative	0-5/HPF	cells/HPF
Urine nitrite	Negative	Negative	NA
Urine leukocyte esterase	Negative	Negative	NA
Urine culture	No growth	No growth or <10K CFU	NA

**Figure 1:**
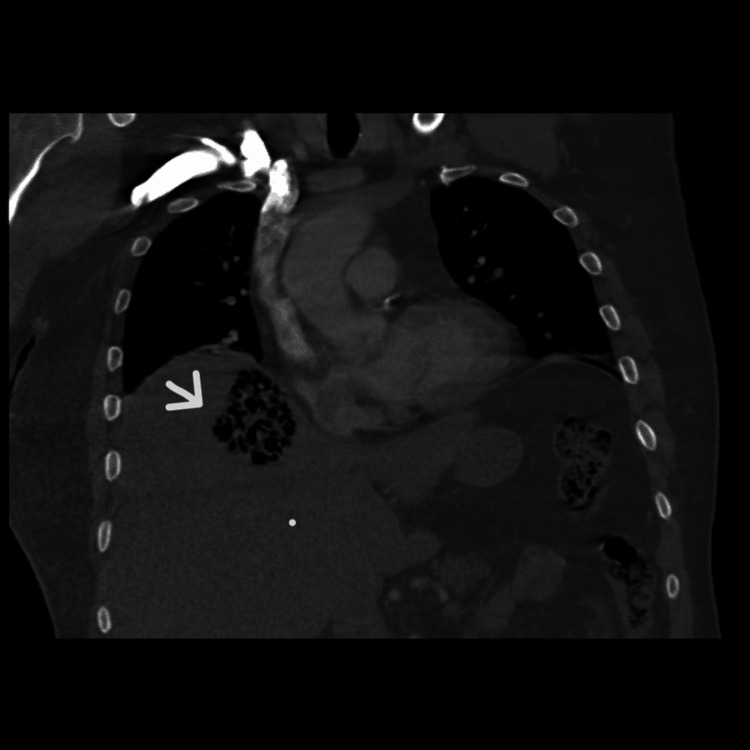
Coronal view of abdominal computerized tomography demonstrating a liver abscess (white arrow)

**Figure 2:**
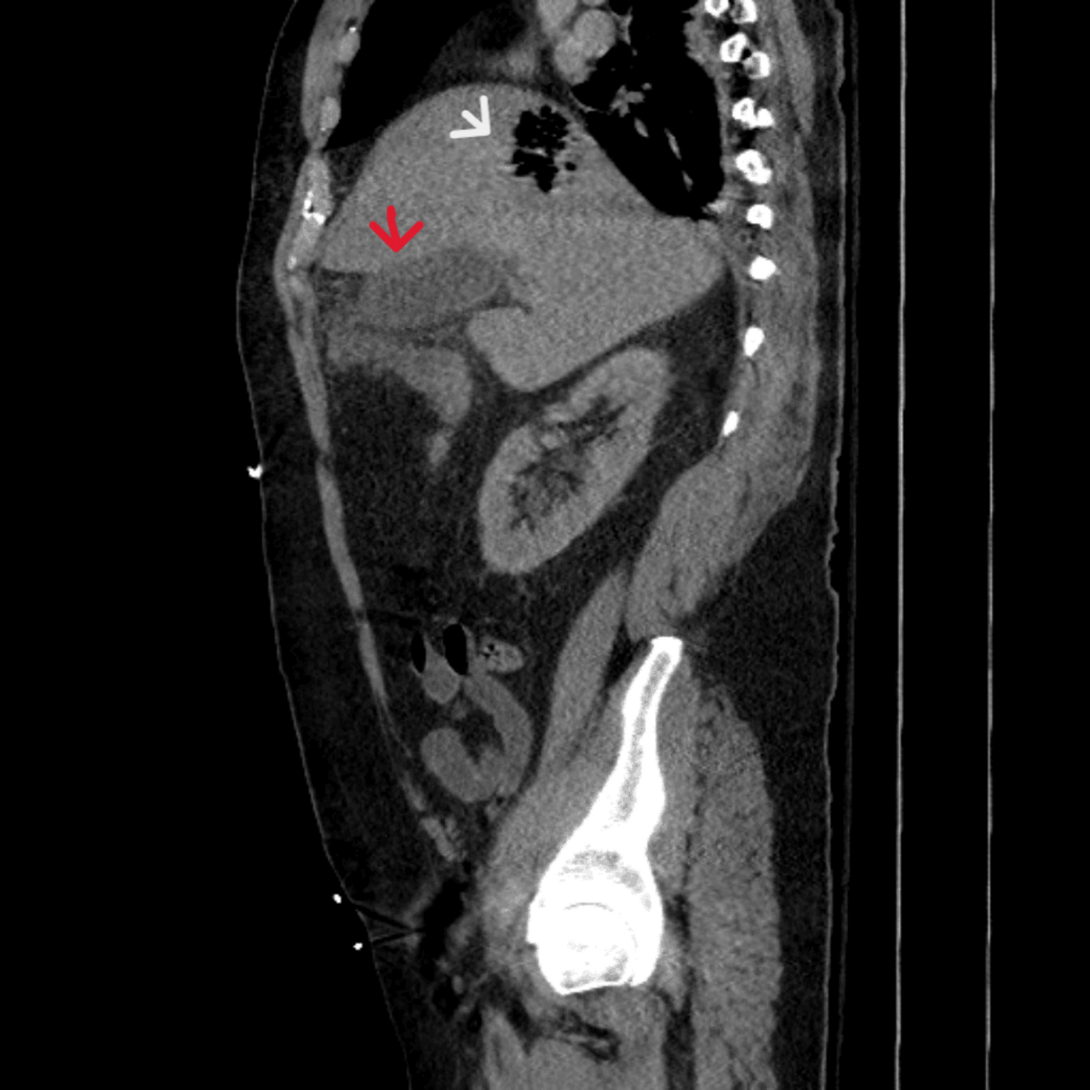
Sagittal view of abdominal computerized tomography demonstrating both a liver abscess (white arrow) and acalculous cholecystitis (red arrow)

A CT scan of the abdomen and pelvis revealed pericholecystic infiltration concerning cholecystitis. No gallstones were identified. There was thickening of the wall of the hepatic flexure of the colon, located immediately adjacent to the gallbladder, which was thought to represent secondary inflammatory involvement. A new collection of mottled gas was identified in the superomedial hepatic dome, measuring 4.0 × 5.0 × 5.8 cm, suggestive of a liver abscess with a gas-forming organism (Figures 1, 2).

The patient was admitted from the ED to the intensive care unit (ICU). Blood cultures were positive for *C. perfringens*. General surgery was consulted due to the findings of a liver abscess and acalculous cholecystitis. The patient subsequently underwent interventional radiology-guided cholecystostomy tube placement. The culture of the abscess drainage also grew *C. perfringens*. Magnetic resonance cholangiopancreatography (MRCP) showed no biliary dilatation or choledocholithiasis. A percutaneous drain was placed by interventional radiology. The patient’s hospital course was complicated by acute kidney injury secondary to acute tubular necrosis, requiring hemodialysis beginning on hospital day 15. The nephrology team attributed the acute kidney injury to bilirubin toxicity from large-volume hemolysis. His renal function improved, and he was discharged to inpatient rehabilitation after 22 days of hospitalization. Two weeks later, the patient underwent a laparoscopic cholecystectomy.

## Discussion

The clinical presentation of *C. perfringens* bacteremia is widely variable. Common features include fever, chills, and malaise.

Leukocytosis may be present, as observed in this case. A definitive diagnosis is established through positive blood cultures [4]. Liver function tests can offer important clues suggestive of underlying hepatic involvement. Hemolysis occurs in approximately 5% to 15% of Clostridium sepsis cases [4]. In this case, abscess aspirate cultures were also positive for *C. perfringens.*

Contrast-enhanced CT of the abdomen and pelvis is an appropriate imaging modality for evaluation. A right upper quadrant ultrasound may also be indicated. This case was notable for the presence of both a hepatic abscess and acalculous cholecystitis.

*C. perfringens* should be considered a potential source of bacteremia in patients with hepatic abscess, gallbladder disease, a recent hepatobiliary procedure, or when imaging reveals a gas-forming organism. Early initiation of antibiotic therapy targeting *C. perfringens* is crucial. Clostridium species are generally susceptible to penicillin G, clindamycin, metronidazole, cefoxitin, and piperacillin/tazobactam [4]. In cases of suspected *C. perfringens* infection, antibiotic regimens should include clindamycin or metronidazole, both of which possess anti-toxin activity.

Rapid source control is essential and may include cholecystectomy or percutaneous drainage of a hepatic abscess. In this patient, large-volume hemolysis was observed and was likely the cause of acute kidney injury, ultimately requiring hemodialysis. Rajendran et al. reported a similar case of intravascular hemolysis and sepsis due to a *C. perfringens* liver abscess, in which the patient also required several days of hemodialysis [7].

Prompt removal of the identifiable source, such as the gallbladder, is indicated when the patient is clinically stable [4]. Surgical drainage may also be necessary.

## Conclusions

*C. perfringens* bacteremia from a hepatic abscess led to hemolysis, causing acute renal injury. *C. perfringens* infections demonstrating hemolysis are rare yet extremely dangerous. The clinical course was further complicated by severe intravascular hemolysis, leading to acute renal failure that required temporary hemodialysis. This case demonstrates the importance of early diagnosis, prompt antibiotic treatment, and timely intervention to manage potential sources.
